# Does medial tourism promote growth in healthcare sector?

**DOI:** 10.1007/s10198-024-01700-3

**Published:** 2024-06-06

**Authors:** Hassan F. Gholipour, Kourosh Esfandiar

**Affiliations:** 1 Western Sydney University, Sydney, Australia; 2 Edith Cowan University, Joondalup, Australia

**Keywords:** Medical tourism, Economic growth, Healthcare sector, Tourism revenue, Panel data

## Abstract

The purpose of this study is to investigate the effect of medical tourism revenues on the growth of healthcare sector across 49 emerging and developed economies from 2008 to 2022. Using panel GMM and PMG/ARDL estimation methods, the results show that higher levels of medical tourism revenues promote growth in the healthcare sector. This finding remains robust across different sample periods, alternative measure of healthcare sector performance, and model specifications.

## Introduction

This study examines the impact of medical tourism revenues on the growth of healthcare sector across both emerging and advanced economies over time. “Medical tourism is a type of tourism activity which involves the use of evidence-based medical healing resources and services (both invasive and non-invasive). This may include diagnosis, treatment, cure, and prevention and rehabilitation” [1]. In recent years, health tourism encompassing both wellness and medical tourism, has experienced significant growth, and has become increasingly relevant in many established and emerging destinations worldwide [2–4]. This growth has been evident through the surge in media coverage and discussions in public policy forums [2, 5].

According to Euromonitor International [6], global medical tourism revenues have significantly increased from USD 24,175.9 million (with 2023 prices) in 2009 to USD 27,931.5 million in 2022, representing a compound annual growth rate (CAGR) of 4.1%. While the sector faced substantial challenges during the COVID-19 pandemic in 2020, it demonstrated a rapid recovery in 2021 and 2022, indicating its robust resilience.

The potential for improved well-being and medical treatment has become a motivating factor for travel [7, 8]. The medical tourism industry is rapidly expanding, with governments recognizing its potential for sustainable growth [9, 10]. Governments in popular medical tourism destinations have implemented policies and strategies to attract international patients. These strategies include investing in healthcare infrastructure, enhancing the quality of medical services, and promoting medical tourism through marketing campaigns. For instance, Thailand and Malaysia have established themselves as leading medical tourism hubs by offering high-quality healthcare services at competitive prices, coupled with their reputation for hospitality and exotic tourist attractions [11–14].

Additionally, the integration of advanced technology in medical procedures and telemedicine as well as the incorporation of artificial intelligence (AI) in medical tourism has further boosted the appeal of medical tourism. Patients can now access preliminary consultations and follow-up care through digital platforms, making the entire process more convenient and efficient. This technological advancement not only improves patient experience but also allows healthcare providers to maintain a continuum of care for international patients [15, 16]. In a comprehensive report, The UNWTO and ETC [1] explores factors contributing to the growth of medical tourism and the health sector. It highlights a confluence of drivers, including increased leisure time and affordable travel, facilitated by accessible information on the internet. Moreover, increased awareness of health issues has driven a greater dedication of resources to healthcare. Overburdened healthcare systems and rising long-term care costs due to aging populations are generating medical tourism, where people seek medical care in other countries. Urbanization of the global population, along with associated factors such as chronic diseases, is leading to a growing need and demand for health-oriented travel experiences. Rapid technological advances, such as less invasive medical technologies, are influencing travel for health purposes. Additionally, v and policies have emerged as influential catalysts, boosting the growth of both domestic and international health tourism. The report also highlights the potential of health tourism to mitigate the seasonality challenges i.e. its adaptability to year-round destination strategies presents a promising avenue for tourism dispersal.

In supporting the report by UNWTO & ETC [1] which was earlier discussed, there is a body of theoretical and empirical evidence indicating that medical tourism—and health tourism more generally—can promote the economic activities in host countries [17]. For example, Beladi et al. [18] examined cross-sectional data from 47 countries over 2007–2013 to test two opposing hypotheses regarding the impact of medical tourism on economic growth. On the positive side, they argued that medical tourism directly contributes to the economic growth of host economies through revenue generated in the tourism sector. This happens as the influx of medical tourists contributes to the local economy by increasing demand for accommodation, transportation, and other tourism-related services. On the negative side, they argued that the development of medical tourism may crowd out medical resources in the public health care sector of the host country, potentially leading to reduced productivity among workers and subsequently reduced access to public healthcare. However, their results showed that medical tourism has, on average, a positive impact on host economies’ growth of gross domestic product (GDP) per capita, particularly in non-OECD countries. They further noted that implementing policies such as taxing medical tourism and using the revenue to support affected sectors can enhance the welfare of the host country, increase the wages of healthcare workers, and contribute to the retention of healthcare professionals in the public health system. Cuba serves as a successful case in this regard (see [19] for details). Likewise, Nola and Radovčić [20] highlighted that the growing medical tourism industry had positive impacts on healthcare accessibility.

Following the above, medical tourism has significant economic implications for both destination and departure countries. It generates hard currency inflows, particularly from higher-income to lower- and middle-income countries, supporting their economic development [21]. Additionally, the growth of medical tourism can lead to investments in new infrastructure and stimulate activity in related economic industries [22]. In terms of healthcare, medical tourism can have several positive effects. It can help to alleviate the burden on domestic healthcare systems by encouraging domestic patients to seek medical care in foreign countries [21]. This can free up resources and reduce waiting times for local patients [14]. Medical tourism can also contribute to the development of healthcare facilities and services in destination countries, as providers strive to meet the needs and expectations of international patients [14]. This can lead to improvements in the quality of healthcare and the adoption of advanced medical technologies [14]. Furthermore, the presence of medical tourists can create opportunities for knowledge exchange and collaboration between healthcare professionals from different countries [23]. This can lead to local medical professionals gaining exposure to international best practices and innovative medical techniques [24].

The positive effects of medical tourism on the economy and healthcare sector have been observed in various countries. For example, tourism-led growth in Taiwan has been found to have a stimulating influence across the overall economy, including the healthcare sector [25]. Similarly, in Pakistan, the development of medical tourism was considered a positive contribution to economic growth [26]. This impact is also evident in countries facing controversy and challenges, such as Iran in the Middle East [27, 28], where despite sanctions and healthcare limitations, the country has become a key destination for medical tourism [29, 30]. The revenues from medical tourism have been instrumental in addressing economic deficiencies within the healthcare sector [31].

The expanding medical tourism industry has given rise to international competition. For example, Malaysia, a prominent player in this sector, confronts competition in its endeavours to attract Chinese medical tourists, with several Southeast Asian nations likewise trying to access this market [32]. Finally, in their bibliometrics study, Habibi et al. [33] found that criteria like cost, service quality, national economy, and healthcare system quality impact medical destination choices. Amidst rising competition in medical tourism, enhancing the emotional experience via marketing strategies is vital.

Although the above-mentioned studies provide valuable insights into the impact of medical tourism on economic activities, the direct link between medical tourism revenues and growth in healthcare sector across countries and over a long period has not received much attention in the literature. In addition, earlier studies (e.g., [18]) often examined the relationship between medical tourism and economic growth at an aggregate level, which may not provide a clear picture of the link. This is partly due to endogenous nature of economic growth models, where various factors may simultaneously influence and be influenced by economic growth. Finally, existing studies (e.g., [18]) often employ standard cross-sectional regression, which may suffer from endogeneity issue. In this study, we use panel Generalized Method of Moments (GMM) and Pooled Mean Group-Autoregressive Distributed Lag (PMG-ARDL) estimators, which minimize the potential problem of endogenous explanatory variables and capture the dynamics of growth in healthcare sector. Our study extends the existing but fast-growing literature on medical tourism-economic activities by examining the impact of medial tourism revenues on host countries’ growth in healthcare sector across 49 emerging and advanced economies over 2008–2022. The other attribute of our study that differentiate our paper from the existing studies is that we do not separate domestic and international medical tourism in the analyses. Previous researchers often analyze the positive and negative impacts of international medical tourism on host countries. However, in our study, we use the aggerate medical tourism revenues, which include both domestic and inbound medical tourists.

Our research is not only motivated by the gap in literature but also responds to the call by the UNWTO-ETC (2018) for further analyses on the impacts of medical tourism on wellbeing of local residents in the destinations. Additionally, this study addresses the recommendations of Beladi et al. [18], who, while using cross-sectional data over a short timeframe, suggested utilizing longer time-series data across countries to explore more causal link between medical tourism and economies’ output growth. Panel data offer several advantages over cross-sectional and time-series data, providing more information, greater variability, less collinearity among the variables, more degrees of freedom and increased efficiency [34].

The article proceeds as follows: Sect. "Data and Method" explains the data, variables and the estimation method; Sect. "Results" reports the findings; and the final section concludes the paper.

## Data and method

### Data: scope of the research

This study uses annual data from 2008 to 2022, covering a total of 49 countries. These countries, comprising both developed and emerging economies, were chosen based on the availability of data related to medical tourism revenues. However, the sample represents a wide range of economic development levels, from highly developed economies such as the United States and the United Kingdom to emerging economies, including India and Brazil. Thus, they also exhibit variability in terms of healthcare infrastructure, healthcare policies, and economic conditions.

The countries included in the sample are Argentina, Australia, Austria, Belgium, Brazil, Bulgaria, Canada, Chile, Colombia, Croatia, Czech Republic, Ecuador, Egypt, France, Germany, Greece, Hong Kong, Hungary, India, Ireland, Israel, Italy, Japan, Kenya, Malaysia, Mexico, Morocco, Netherlands, New Zealand, Peru, Philippines, Poland, Portugal, Romania, Russia, Saudi Arabia, Singapore, Slovakia, South Africa, South Korea, Spain, Switzerland, Taiwan, Thailand, Turkey, Ukraine, United Arab Emirates, the United Kingdom and the United States.

### Dependent variable

In this study, the dependent variable is the growth in healthcare sector. As a proxy for this variable, we use the growth in share of gross value added (GVA)^1^ from health and social work (USD million) in total GVA. Data for both aggregate and sectoral GVA were obtained from Euromonitor International [6]. We divide GVA from health and social work by total GVA to have comparable values across larger and smaller economies in our sample. The strongest growth in GVA from health/total GVA over the 2008–2022 was observed in Romania (9.92%) and Vietnam (9.15%). The lowest growth was for Ireland ( − 2.84) and China ( − 3.15%). The descriptive statistics of the variables (before transformation) are presented in Table 1.

**Table 1:** Descriptive statistics (before transformation) for full sample, 2008–2022

	Mean	Standard deviation
GVA_HEALTH	0.020	0.085
MEDICAL	15.887	39.454
GDPC_G	0.016	0.039
GOV_HEALTH	1,705.835	1,747.295
CONS_HEALTH	816.144	1,448.826

GVA_HEALTH is the growth in share of GVA from health sector in total GVA; MEDICAL is medical tourism revenues per capita; GDPC_G is GDP per capita growth (annual %); GOV_HEALTH is government expenditure on health per capita; CONS_HEALTH is consumer expenditure on health goods and medical services per capita

### Variable of interest

In this study, the explanatory variable of interest is countries’ medical tourism. As a proxy for medical tourism activities, we use the medical tourism revenues which are collected by Euromonitor International [6]. It is defined as “*medical tourism value sales concern all domestic and inbound trips that have the purpose of some kind of medical treatment regardless of complexity. Aesthetic or cosmetic surgery is also included. Only expenses for travel and tourism services such as hotels, travel in destination, car rental, intermediaries, *etc.,* in the country are included, while medical expenses are excluded as the focus is on the revenue generated for travel and tourism through medical tourism. Travel services expenses of both the patient and any accompanying people are included. For inbound trips for medical purposes, transport tickets bought in the country of departure are excluded*.”

To account for variations in country size, we normalize the medical tourism revenues (in million US dollars) through dividing the revenues by number of populations, enabling cross-country comparisons. The average of medical tourism revenue per capita in our sample over the period 2008–2022 is USD 15.88, with the highest per capita revenues were recorded in the United Arab Emirates (USD 209.28) and Switzerland (USD 136.01), while the lowest were Kenya (USD 0.173) and Croatia (USD 0.12).

It is noteworthy that the Euromonitor International’s tourism data have become widely accepted and served many researchers in recent years (e.g., [35–37]). However, we acknowledge potential limitations of the Euromonitor International’s tourism data, as this source relies on estimated values for tourism-related variables in some countries and lacks data for many developing countries.

### Control variables

In addition to our main variable of interest (medical tourism), we control for other important determinants of growth in healthcare sector in our panel data modelling. These include economic growth (measured by annual GDP per capita growth rate), government expenditure on health per capita, and consumer expenditure on health goods and medical services per capita. The data for GDP per capita growth (annual %) are obtained from the World Bank [38], while government and consumer health expenditure data are collected from Euromonitor International [6]. The choice of control variables for our model specification is guided by the availability of data for variables across countries and over time. Although the number of control variables are limited due to data availability, we argue that including one-year lag of dependent variable, taking the first difference of variables (which captures unobserved factors in countries), including year fixed effects and controlling for economic growth (which, to a large extent, capturing labour productivity growth and capital formation in the economy) would be sufficient for model specification.

### Estimation method

We apply a dynamic panel model which is estimated by the GMM. The model is useful for our data set because the dynamic panel model is designed for panels with a large number of cross-sections (N = 49) and a short time series (T = 15). Moreover, it is likely that there exists persistence in the dynamics of GVA_HEALTH, such that the previous level of GVA_HEALTH has an influence on the current level. The other reason to use the GMM approach for estimation is to minimize the potential problem of endogenous explanatory variables in Eq. (1). For example, there is the potential issue of reverse causality: the stronger growth in healthcare sector might attract more medical tourists, rather than the other way around. Equation (1) presents our empirical model:

1 $${\text{GVA}}\_{\text{HEALT}}_{{\text{it}}} = \, \beta_{1} {\text{GVA}}\_{\text{HEALT}}_{{\text{i}},{\text{t}} - {1}} + \, \beta_{2} {\text{MEDICAL}}_{{\text{it}}} + \, \delta_{3} {\text{X}}_{{\text{it}}} + \, \Omega_{\text{t}} + {\text{ u}}_{{\text{it}}}$$

where GVA_HEALTH is the growth in share of GVA from health sector in total GVA, GVA_HEALT_i,t-1_ is the lagged GVA_HEALTH, MEDICAL is medical tourism revenues per capita, X_it_ is a vector that includes the control variables, *i* = 1,…, n denotes the country, *t* = 1, …, t denotes the time period, *Ω*_*t*_ takes into account the time effect and *u*_*it*_ is an error term.

All variables are converted into logarithmic form for the empirical estimation with the exception of GVA_HEALTH and GDPC_G. Two lags of dependent variable and one lag of explanatory variables are used as instruments in the GMM estimations for main analyses.

## Results

### Main analyses

The results of the GMM estimator are presented in Table 2, columns 1–4. Column 1 includes one lag of the dependent variable and variable of interest (medical tourism revenues) as explanatory variables for GVA_HEALTH. The results show that MEDICAL has the expected positive association with GVA_HEALTH and is statistically significant at the 1% level, indicating that higher levels of medical tourism revenues per capita promote stronger growth in healthcare sector in our sample countries. This finding supports our hypothesis that we put forward. It is also in line with what several previous empirical studies reported that medical tourism has a positive impact on economic activities (e.g., [39]) and in particular in health sector (e.g., [14]).

**Table 2:** Results of GMM estimator

Dependent variable: GVA_HEALTH				
	*Without control variables*	*With control variables*		
*Explanatory variable*	(1)	(2)	(3)	(4)
GVA_HEALTH (-1)	− 0.390*** (0.004)	− 0.393*** (0.004)	− 0.414*** (0.004)	− 0.411*** (0.004)
MEDICAL	0.066*** (0.020)	0.067*** (0.021)	0.067*** (0.018)	0.045** (0.021)
GDPC_G		− 0.005 (0.077)	0.083 (0.056)	0.040 (0.074)
GOV_HEALTH			− 0.195*** (0.021)	− 0.229*** (0.038)
CONS_HEALTH				0.154* (0.086)
Year dummies	Included	Included	Included	Included
*p*-value of AR (2)	0.570	0.426	0.289	0.137
*p*-value of Sargan test	0.340	0.235	0.350	0.416
Total panel observations	471	460	438	438

* 0.10, ** 0.05, *** 0.01. Standard errors are in parentheses. GVA_HEALTH is the growth in share of GVA from health sector in total GVA; MEDICAL is medical tourism revenues per capita; GDPC_G is GDP per capita growth (annual %); GOV_HEALTH is government expenditure on health per capita; CONS_HEALTH is consumer expenditure on health goods and medical services per capita

In columns 2–4, we insert other possible determinants of growth in healthcare sector in the model. However, including these additional explanatory variables does not change the positive and significant association between MEDICAL and GVA_HEALTH. The results also show that the coefficient of one-year lag of dependent variable (GVA_HEALTH (-1)) is negative and significant meaning that countries that have experienced stronger growth in the healthcare sector in the past year have slower growth in the current year. We also find that while government expenditure on health per capita (GOV_HEALTH) has a negative impact on GVA_HEALTH, consumer expenditure on health goods and medical services per capita (CONS_HEALTH) has a positive influence on GVA_HEALTH. The negative relationship between GOV_HEALTH and GVA_HEALTH can be explained by the crowd-out effect. This concept suggests that increased government involvement in healthcare limits opportunities for the private sector to expand, consequently impeding overall health sector growth [40]. In other words, elevated government healthcare expenditure can constrain the development of private health entities, ultimately stifling the health sector’s overall progress.

Two diagnostic tests of the GMM estimations are reported at the bottom of Table 2. The *p*-values of chi square of Sargan test for over-identifying restrictions and the Arellano–Bond test for second order correlation (AR (2)) in the first-differenced errors are not significant at the 5% level. It means that the instruments are valid and there is no serial correlation in the models.

### Robustness checks

This section details three robustness checks performed to confirm the validity of our main findings, which are explained in the following sections.

*Removing outlier years*: We exclude the years 2008–2009 and 2020–2022 from our sample, focusing only on the period from 2010 to 2019. This exclusion is necessary because the global economy and tourism sector experienced substantial disruptions during these periods due to the Global Financial Crisis and the COVID-19 pandemic [41, 42]. Therefore, 2008–2009 and 2020–2022 are considered outliers in our analysis. The results from this robustness test are similar to those of the full sample regressions (see column 1 of Table 3). The coefficient of medical tourism revenues has the expected positive sign and is statistically significant.

**Table 3:** Results of robustness checks: Excluding crisis years & alternative estimator

Dependent variable: GVA_HEALTH		
	Sample 2010–2019 GMM estimator	Sample 2008–2022 PMG/ARDL estimator
*Explanatory variable*	(1)	(2)
GVA_HEALTH (-1)	− 0.441*** (0.003)	
MEDICAL	0.034** (0.015)	0.009*** (0.002)
GDPC_G	0.251*** (0.078)	− 0.414*** (0.066)
GOV_HEALTH	− 0.291*** (0.048)	0.004 (0.003)
CONS_HEALTH	0.533*** (0.087)	− 0.005 (0.004)
Year dummies	Included	
*p*-value of AR (2)	0.707	
*p*-value of Sargan test	0.427	
COINTEQ		− 0.931*** (0.063)
Total panel observations	360	487

* 0.10, ** 0.05, *** 0.01. Standard errors are in parentheses. Selected Model for PMG/ARDL estimation: ARDL(1, 1, 1, 1, 1). GVA_HEALTH is the growth in share of GVA from health sector in total GVA; MEDICAL is medical tourism revenues per capita; GDPC_G is GDP per capita growth (annual %); GOV_HEALTH is government expenditure on health per capita; CONS_HEALTH is consumer expenditure on health goods and medical services per capita

*Applying panel PMG/ARDL estimator*: In addition to GMM estimation, we also apply panel PMG/ARDL estimator [43] to examine the long-run relationship between the medical tourism revenues and the growth in health sector across countries and over time. In recent years, an increasing number of researchers in the field of tourism have employed this estimation method (e.g., [44, 45]).

According to Pesaran and Smith [46], Pesaran [47], and Pesaran et al. [43], the ARDL method is useful for long-run analysis, and its methodology is valid regardless of whether the regressors are exogenous, or endogenous, and irrespective of whether the underlying variables are I(0) or I(1) [48]. The pooled mean group (PMG) estimator “allows the intercepts, short-run coefficients, and error variances to differ freely across groups, but constrains the long-run coefficients to be the same” [43], p.621). We estimate the following ARDL (*p*, *q*, *q*,…, *q*) model:

2 $$GVA\_HEALTH_{it} = \mathop \sum \limits_{j = 1}^p \lambda_{ij } GVA\_HEALTH_{i,t - j} + \mathop \sum \limits_{j = 0}^q \delta_{ij}^{\prime} x_{i,t - j} + \mu_i + \varepsilon_{it}$$

where *t* = 1, 2, …, T, and countries, *i* = 1, 2, …, N, x_it_ (*k* × l) is the vector of explanatory variables for country *i*; ${\mu }_{i}$ represent the fixed effects; the coefficients of the lagged dependent variables,${\lambda }_{ij}$, are scalars; and ${\delta }_{ij}$ are *k* × l coefficient vectors.

Column 2 of Table 3 reports the long-run results obtained using the PMG/ARDL estimator. In line with our main findings in Table 2, medical tourism revenues (MEDICAL) is positively and significantly related to the growth in healthcare sector (GVA_HEALTH).

*Alternative proxies for performance of healthcare sector:* For another robustness check, we re-estimate Eq. 1, replacing the original dependent variable of healthcare sector growth (GVA_HEALTH) with competitiveness of healthcare sector (COMPET_HEALTH). As a proxy for COMPET_HEALTH, we use the World Economic Forum (WEF)’s Global Competitiveness Index: Health. This index is the fifth pillar of the WEF’s aggregate index and is categorized under Human Capital sub-dimension. The health index ranges from 0 (lowest) to 100 (highest) and measures the national competitiveness of the health sector. Data for this variable are obtained from Euromonitor International [6] database. France (98.64), Singapore (98.25) and Italy (97.61) have the most competitive health sector whereas India (56.03), Kenya (54.32) and South Africa (39.37) have the lowest scores in this index among sample countries. The data for this analysis includes the period of 2008–2019 as data for COMPET_HEALTH are available till 2019. The GMM estimation method is used for the analysis, and the results are presented in Table 4. The coefficient of MEDICAL maintains its positive sign and is statistically significant at 1% level. A one percent increase in medical tourism revenues per capita leads to 0.035% rise in WEF’s Global Competitiveness Index: Health. In general, this finding reaffirms our earlier results obtained in Tables 2 and 3 where we employed GVA_HEALTH as a measure of development in healthcare sector.

**Table 4:** Results of robustness checks: Alternative proxy for healthcare performance

Dependent variable: COMPET_HEALTH	
*Explanatory variable*	Sample 2008–2019 GMM estimator
COMPET_HEALTH ( − 1)	0.435*** (0.035)
MEDICAL	0.035*** (0.011)
GDPC_G	− 0.110*** (0.026)
GOV_HEALTH	0.019* (0.011)
CONS_HEALTH	− 0.007 (0.016)
Year dummies	Included
*p*-value of AR (2)	0.430
*p*-value of Sargan test	0.447
Total panel observations	447

* 0.10, ** 0.05, *** 0.01. Standard errors are in parentheses. COMPET_HEALTH is logarithm of the WEF’s Global Competitiveness Index: Health; MEDICAL is medical tourism revenues per capita; GDPC_G is GDP per capita growth (annual %); GOV_HEALTH is government expenditure on health per capita; CONS_HEALTH is consumer expenditure on health goods and medical services per capita. One-year lag of dependent and explanatory variables are used as instruments

## Discussion and conclusion

While there have been a number of conceptual and cross-sectional studies investigating the association between medical tourism and economic outputs, no empirical research has examined the relationship between medical tourism revenues and growth in the healthcare sector using the recent data across developed and emerging economies. Using data from 49 developed and emerging economies over 2008–2022, we find that medical tourism revenues per capita has a positive impact on growth in healthcare sector (measured by the GVA from healthcare sector/overall GVA).

The findings of this study are in line with previous research. For example, Türedi et al. [49] explored the relationship between healthcare sector development and inbound tourism in ASEAN countries from 2000 to 2018. Their investigation revealed a long-term (cointegrating) association between inbound tourism and healthcare sector development. This indicates a positive connection between revenues generated from medical tourism and the expansion of the healthcare sector. The revenue obtained from medical tourism services often spills over to the secondary and tertiary sectors. This suggests that the revenues generated from medical tourism can contribute to the overall growth of the healthcare sector. An illustrative example from earlier studies is that of Johnston et al. [21], who highlighted the case of Cuba, which has established itself as a destination for medical tourism and has used the revenue generated from this industry to fund its public healthcare system, demonstrating a direct link between medical tourism revenues and the expansion of the healthcare sector. Similarly, Beladi et al. [18] confirmed that medical tourism positively impacts economic growth in non-OECD countries by generating substantial revenue. This revenue enhances healthcare services, improves welfare, increases healthcare workers' wages, retains skilled medical workers, and upgrades healthcare infrastructure. Hazarika [50] detailed the rapid growth of India's healthcare sector driven by medical tourism. The influx of international patients has generated substantial revenue, leading to increased investments in healthcare infrastructure, technologies, and specialized services. This revenue is being reinvested to further develop India's private healthcare sector. However, Hazarika cautions that the benefits of medical tourism should not be confined to the private sector. To ensure equitable distribution of gains, investments in the public health system must also be increased. This approach would enhance the overall healthcare system, ensuring that both private and public sectors benefit from medical tourism.

In conclusion, the evidence supports the claim that there is a positive correlation between revenues generated from medical tourism and the growth and competitiveness of the healthcare sector. Thus, the financial resources generated through medical tourism can contribute to economic development, fund public healthcare systems, and promote healthcare sector expansion. However, it is important to consider two points raised in some of these studies. Firstly, greater government financial support is essential. Therefore, in light of the empirical findings presented in this research, it is recommended that policymakers allocate adequate financial resources to enhance medical services, aligning with the guidance provided by Béland and Zarzeczny [51]. They emphasized the importance of improving the institutional aspects of national healthcare systems, particularly for destinations seeking to bolster their tourism industry. This holds particular significance given that many countries must adapt their healthcare systems to meet emerging challenges, including the rise of medical tourism. Secondly, the issue of unequal access to healthcare for local and regional populations or those with lower incomes as a consequence of the expansion of medical tourism is highlighted. As Connell [52] noted, medical tourism may pose challenges to local healthcare providers. This is evident in countries like India, where the migration of healthcare workers to hospitals focusing on medical tourism has disadvantaged regional areas [53]. On the other hand, some countries, such as Singapore, have effectively developed an equitable healthcare system [54, 55].

This study is not without limitations, one of which relates to its exclusive focus on both emerging and developed economies. Expanding the scope of investigation to encompass other regions, particularly underdeveloped countries or concentrating on specific areas such as the Organisation of Islamic Cooperation (OIC) and especially Asia–Pacific which has gained popularity as a preferred destination for global medical travellers due to its medical expertise, technological advancements, safety standards, tourism appeal, and cost advantages [56], would be advantageous. Future research should also explore how medical tourism can impact public resources and potentially causes health inequities. Finally, future studies could examine the impact of domestic versus foreign medical tourism expenditures on the growth of the health sector, provided that data for both indicators become available over time and across countries.

## Footnotes

1 According to Euromonitor International [6], GVA is “the value of output less the value of intermediate consumption. It is calculated without making deductions for depreciation of fabricated assets or for depletion and degradation of natural resources.” In other words, “GVA is a measure of the contribution to gross domestic product (GDP) made by an individual producer, industry or sector.”.
